# Supplementary material for: Hybrid Models and Biological Model Reduction with PyDSTool
Source: PLoS Comput Biol. 2012 Aug 9;8(8):e1002628. doi: 10.1371/journal.pcbi.1002628 (PMC3415397; doi:10.1371/journal.pcbi.1002628)
Supplement: Text S4 — Complete source code for the PyDSTool package (version 0.88.120504). Includes API documentation and help files linking to web pages. This file is identical to the current public release on Sourceforge.net. (ZIP) [file pcbi.1002628.s004.zip › PyDSTool/html/PyDSTool.Toolbox.ParamEst.BoundMin-class.html]

xml version="1.0" encoding="ascii"?


PyDSTool.Toolbox.ParamEst.BoundMin


| Home | Trees | Indices | Help | PyDSTool |
| --- | --- | --- | --- | --- |

Package PyDSTool ::
Package Toolbox ::
Module ParamEst ::
Class BoundMin

[hide private]

[frames] | no frames]

# Class BoundMin

source code

```
    object --+        
             |        
common.Utility --+    
                 |    
          ParamEst --+
                     |
                    BoundMin
```

---

Bounded minimization parameter and initial condition optimizer for
one-dimensional DS trajectories. Fits 1 parameter only.

Uses SciPy.optimize fminbound algorithm.


|  |  |
| --- | --- |
| Instance Methods | [hide private] |

|  |  |
| --- | --- |
| \_\_init\_\_(self, \*\*kw)  x.\_\_init\_\_(...) initializes x; see x.\_\_class\_\_.\_\_doc\_\_ for signature | source code |

|  |  |
| --- | --- |
| run(self, parConstraints, xtol=5e-05, maxiter=500, extra\_args=`(``)`, verbose=False)  Run parameter estimation. | source code |

**Inherited from `ParamEst`**:
`evaluate`,
`find_logs`,
`iterate`,
`key_logged_residual`,
`par_sensitivity`,
`pars_array_to_dict`,
`pars_dict_to_array`,
`pars_to_ixs`,
`resetParArgs`,
`reset_log`,
`setAlgParams`,
`setFn`,
`show_log_record`,
`weighted_par_sensitivity`

**Inherited from `object`**:
`__delattr__`,
`__getattribute__`,
`__hash__`,
`__new__`,
`__reduce__`,
`__reduce_ex__`,
`__repr__`,
`__setattr__`,
`__str__`


|  |  |
| --- | --- |
| Properties | [hide private] |

**Inherited from `object`**:
`__class__`


|  |  |
| --- | --- |
| Method Details | [hide private] |

|  |  |
| --- | --- |
| \_\_init\_\_(self, \*\*kw)  *(Constructor)* | source code |

x.\_\_init\_\_(...) initializes x; see x.\_\_class\_\_.\_\_doc\_\_ for
signature

Overrides: object.\_\_init\_\_: *(inherited documentation)*

|  |  |
| --- | --- |
| run(self, parConstraints, xtol=5e-05, maxiter=500, extra\_args=`(``)`, verbose=False) | source code |

Run parameter estimation. Returns a dictionary:

'success' -> boolean 'pars\_sol' -> fitted values of pars
'pars\_orig' -> original values of optimized pars 'sys\_sol' ->
trajectory of best fit Model trajectory 'alg\_results' -> all other
algorithm information (list)

Overrides: ParamEst.run: *(inherited documentation)*

  


| Home | Trees | Indices | Help | PyDSTool |
| --- | --- | --- | --- | --- |

|  |  |
| --- | --- |
| Generated by Epydoc 3.0.1 on Fri May 4 15:24:08 2012 | http://epydoc.sourceforge.net |
